# Beware of Green Flounces

**Published:** 1858-01

**Authors:** 


*Beware of Green Flounces.*—We have always been aware that a certain "killing" power is generally conceded to young ladies eyes. It appears that their dress too may possess the same property without a metaphor. In a recent French chemical journal, a member of the Council of Salubrity informs the public that a lady having purchased some green gauze for a ball dress, sent it to be made up, but that five of the seamstresses employed on it were taken ill during the progress of the work. Samples of the gauze being sent to M. Payen, the chemist, it was discovered that the gauze in question received its delicate tint from Schweinfurth green, a pigment composed of arsenite and acetate of copper. This does not adhere firmly to the material, so that the people who made that beautiful fabric, the shopman who sold it, and the women who made it up, all laid themselves liable to disease if not to death, by the incautious handling of it.

It is not the most agreeable sensation in the world, when you are whirling your lovely partner through the mazes of the waltz, or piloting her through the billowy motions of the cotillion, that all the while, in return for your attentions, her animated and graceful motions may be whirling arsenical dust up your nostrils. We cannot be too hard upon the unconscious Brinvilliers, but, nevertheless, to all gay young men, we repeat the friendly warning, "*beware of green flounces.*"

